# Innovative molecular diagnosis of *T* *richinella* species based on β‐carbonic anhydrase genomic sequence

**DOI:** 10.1111/1751-7915.12327

**Published:** 2015-12-07

**Authors:** Reza Zolfaghari Emameh, Marianne Kuuslahti, Anu Näreaho, Antti Sukura, Seppo Parkkila

**Affiliations:** ^1^ School of MedicineUniversity of TampereFI‐33520TampereFinland; ^2^ BioMediTechUniversity of TampereFI‐33520TampereFinland; ^3^ Fimlab Laboratories Ltd and Tampere University HospitalBiokatu 4FI‐33520TampereFinland; ^4^ Department of Veterinary Biosciences, Faculty of Veterinary MedicineUniversity of HelsinkiFI‐00014HelsinkiFinland

## Abstract

Trichinellosis is a helminthic infection where different species of *T* *richinella* nematodes are the causative agents. Several molecular assays have been designed to aid diagnostics of trichinellosis. These assays are mostly complex and expensive. The genomes of *T* *richinella* species contain certain parasite‐specific genes, which can be detected by polymerase chain reaction (PCR) methods. We selected β‐carbonic anhydrase (*β‐* *CA*) gene as a target, because it is present in many parasites genomes but absent in vertebrates. We developed a novel β‐CA gene‐based method for detection of *T* *richinella* larvae in biological samples. We first identified a β‐CA protein sequence from *T* *richinella spiralis* by bioinformatic tools using β‐CAs from *C* *aenorhabditis elegans* and *D* *rosophila melanogaster*. Thereafter, 16 sets of designed primers were tested to detect β‐CA genomic sequences from three species of *T* *richinella*, including *T* *. spiralis*, *T* *richinella* *pseudospiralis* and *T* *richinella* *nativa*. Among all 16 sets of designed primers, the primer set No. 2 efficiently amplified β‐CA genomic sequences from *T* *. spiralis*, *T* *. pseudospiralis* and *T* *. nativa* without any false‐positive amplicons from other parasite samples including *T* *oxoplasma gondii*, *T* *oxocara cati* and *P* *arascaris equorum*. This robust and straightforward method could be useful for meat inspection in slaughterhouses, quality control by food authorities and medical laboratories.

## Introduction

*Trichinella* spp. are the causative agents of a zoonotic helminthic disease, which is called trichinellosis (Gottstein *et al*., 2009). Within the main sources of *Trichinella* spp. [including pigs, horses, wild boars, dogs, walruses, foxes, bears and birds (Alban *et al*., 2011)], the most important source of human infection worldwide is the domestic pig, e.g. in Europe, whereas horse and wild boar meat have played a significant role during outbreaks within the past three decades (Gottstein *et al*., 2009). Infection in humans occurs with the ingestion of insufficiently cooked meat contaminated with *Trichinella* larvae, which are encysted in muscle tissues of the host animals (Clausen *et al*., 1996; Pozio, 2007; Gottstein *et al*., 2009). Due to the re‐emerging problem also in Europe, the European Union and some associated non‐European Union member countries still run *Trichinella* monitoring programmes (Gottstein *et al*., 2009). Following delivery by the gravid female worm, which lives within the intestinal mucosa of the host, newborn larvae (NBL) predominantly migrate into lymphatic and blood vessels of the host and thereafter to the ultimate target organ, i.e. highly oxygenated muscle. NBL enter the striated muscle cells by the aid of their stylet (Gottstein *et al*., 2009). NBL survive in muscle nurse cells for years (up to 40 years in humans and over 20 years, e.g. in polar bears) (Froscher *et al*., 1988; Kumar *et al*., 1990). Among 12 different genetically detected *Trichinella* spp., *Trichinella spiralis* is the most common species in domestic and wild swine and is also the most important aetiological agent that causes wide and global distribution of trichinellosis in humans (Pozio and Darwin Murrell, 2006). Other *Trichinella* spp. include *Trichinella nativa* (La Rosa *et al*., 2001; Nelson *et al*., 2003), *Trichinella pseudospiralis* (La Rosa *et al*., 2001; Pozio and Darwin Murrell, 2006), *Trichinella britovi* (Gottstein *et al*., 2009), *Trichinella murelli* (Ancelle, 1998), *Trichinella nelsoni* (Marucci *et al*., 2009), *Trichinella papuae* (Pozio *et al*., 2005), *Trichinella zimbabwensis* (Pozio *et al*., 2002), *Trichinella* genotype T8 (Marucci *et al*., 2009), *Trichinella* genotype T9 (Zarlenga *et al*., 2006) and *Trichinella* genotype T12 (Krivokapich *et al*., 2012). Seven percent of infected animals carry mixed infections (Airas *et al*., 2010).

Diagnosis of trichinellosis can be made by several methods. The classical method includes meat inspection and sampling for direct microscopic detection of larvae (Nockler *et al*., 2000). The other choices are detection of anti‐*Trichinella* immunoglobulin G (IgG) in animal serum or meat juice by serological methods including enzyme‐linked immunosorbent assay (Gamble *et al*., 2000; Korinkova *et al*., 2008), proteomics (Wang *et al*., 2014), and molecular methods including conventional polymerase chain reaction (PCR), real‐time PCR, loop‐mediated isothermal amplification (Tantrawatpan *et al*., 2012; Lin *et al*., 2013) and reverse line blot hybridization (Rombout *et al*., 2001). Many studies have been focused on detection of novel molecular markers, because the classical methods have limitations, such as labour‐intensiveness, necessity of high level expertise for direct microscopic diagnosis, and high costs due to the required manpower. The potential molecular markers have included Internal transcribed spacer (ITS1 and ITS2) (Zarlenga *et al*., 1999; Lin *et al*., 2013), mitochondrial cytochrome C oxidase subunit III (cox3) gene (Van De *et al*., 2012), mitochondrial large subunit ribosomal RNA (rrnL) (Borsuk *et al*., 2003; Guenther *et al*., 2008; Blaga *et al*., 2009; Li *et al*., 2011), mitochondrial small subunit ribosomal RNA (rrnS) (Blaga *et al*., 2009), DNA sequence of the 5S rRNA intergenic spacer regions (Rombout *et al*., 2001), migratory DNA from *Trichinella* larvae (Li *et al*., 2010) and aminopeptidase (TsAP) gene (Zhang *et al*., 2013).

One effective strategy for specific detection of *Trichinella* infection is identification of a genomic or proteomic determinant, which is absent in host genomes or proteomes (Duplessis and Moineau, 2001). Beta carbonic anhydrase (β‐CA) could be one of such host‐specific markers (Fasseas *et al*., 2010; Syrjanen *et al*., 2010; Zolfaghari Emameh *et al*., 2014; 2015). Six evolutionary divergent gene families encode for CAs (α, β, γ, δ, ζ and η) (Lane *et al*., 2005; Xu *et al*., 2008; Ferry, 2010; Del Prete *et al*., 2014; Zolfaghari Emameh *et al*., 2014). Different CA isozymes are involved in many biological processes, such as respiration involving transport of CO_2_ and bicarbonate between metabolizing tissues, pH homeostasis, electrolyte transfer, bone resorption, and calcification. Moreover, they participate in some biosynthetic reactions, such as gluconeogenesis, lipogenesis and ureagenesis in animals (Vullo *et al*., 2004; 2005; Alterio *et al*., 2006; Nishimori *et al*., 2007; Supuran, 2008), growth of some bacterial species (Merlin *et al*., 2003; Mitsuhashi *et al*., 2004) and photosynthesis in plants and algae (Fawcett *et al*., 1990; Funke *et al*., 1997). The previous investigations have confirmed that *β‐CA* gene sequences are completely absent in vertebrate genomes, whereas they can be found in plants, algae, yeasts, bacteria, archaea, protozoa and invertebrate metazoans (Fawcett *et al*., 1990; Funke *et al*., 1997; Smith *et al*., 2000; Merlin *et al*., 2003; Mitsuhashi *et al*., 2004; Aguilera *et al*., 2005; Fasseas *et al*., 2010; Syrjanen *et al*., 2010; Zolfaghari Emameh *et al*., 2014). By this background, we hypothesized that genomic *β‐CA* sequences from prokaryotic pathogens and eukaryotic parasites would be an ideal host‐specific determinant for molecular diagnostics, with minimal interfering effects from mammalian genomes.

In this study, we designed and piloted a PCR method for rapid detection of three species of *Trichinella* larvae, including *T. spiralis*, *T. pseudospiralis* and *T. nativa* in meat samples.

## Results

### Multiple sequence alignment (MSA)

**Figure 1:**
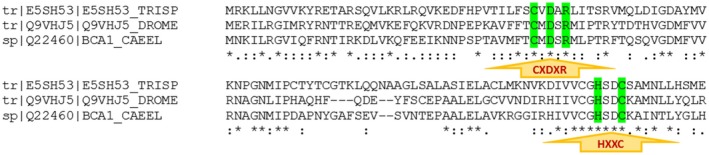
Multiple sequence alignment (MSA) of β‐CA protein sequences from *D* *. melanogaster* (UniProt ID: Q9VHJ5), *C* *. elegans* (UniProt ID: Q22460) and *T* *. spiralis* (UniProt ID: E5SH53). The first (CxDxR) and second (HxxC) highly conserved residues are shown by arrows and marked with green colour.

MSA of the β‐CA protein sequence from *T. spiralis* (UniProt ID: E5SH53) against the sequences from *Drosophila melanogaster* (UniProt ID: Q9VHJ5) and *C* *aenorhabditis elegans* (UniProt ID: Q22460) revealed that they all contain the first (CxDxR; C: cystein, D: aspartic acid, R: arginine, and x: any residue) and second (HxxC; H: histidine, C: cystein, and x: any residue) highly conserved residues (Fig. 1). Hence, the β‐CA protein sequence from *T. spiralis* (UniProt ID: E5SH53) extracted correctly through the Basic Local Alignment Search Tool (BLAST) homology analysis and contained identical highly conserved residues to the predefined β‐CA protein sequences.

### Primer design and PCR

**Figure 2:**
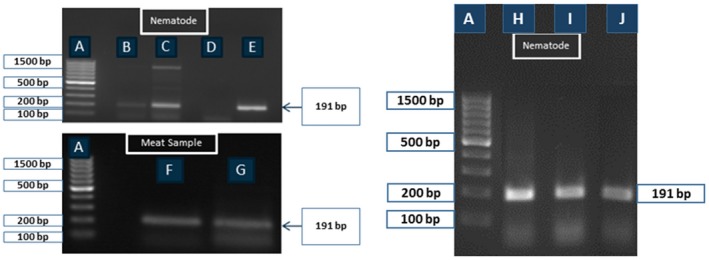
PCR‐based detection of genomic β‐CA sequence from *T* *richinella* larvae and infected meat samples. The primer set No. 2 showed a clear PCR product (size = 191 kb) in all *T* *. spiralis*‐positive samples. (A) 100 bp ladder; (B) one larva; (C) five larvae; (D) negative control (PCR reaction mixture without digested sample); (E) more than 10 larvae; (F and G) 5 mg of *T* *. spiralis* infected mouse meat samples; (H) *T* *. nativa* (10 larvae); (I) *T* *. pseudospiralis* (10 larvae); (J) *T* *. spiralis* (10 larvae).

**Figure 3:**
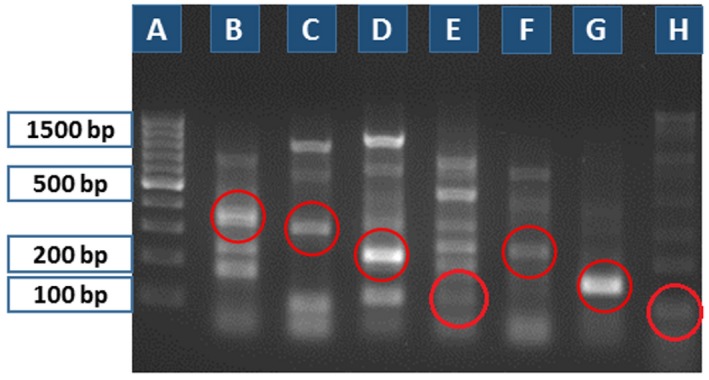
Second round of PCR‐based diagnosis tested on *T* *. nativa* and *T* *. pseudospiralis* samples (10 larvae/each lane). Primer set Nos. 5–7 for *T* *. pseudospiralis* and primer set Nos. 8–11 for *T* *. nativa*. (A) 100 bp ladder; (B) primer set No. 8 (372 bp), (C) primer set No. 9 (317 bp), (D) primer set No. 10 (226 bp), (E) primer set No. 11 (116 bp) for *T* *. nativa*; and (F) primer set No. 5 (251 bp), (G) primer set No. 6 (158 bp), (H) primer set No. 7 (103 bp) for *T* *. pseudospiralis*. The relevant PCR products are shown in red circles.

Among the four different pairs of primers (Nos. 1–4) only the primer set No. 2 (PCR product size 191 bp) showed a clear positive DNA band on agarose gel electrophoresis for detection of *T. spiralis* β‐CA genomic sequence in larvae and meat samples (data not shown). It also produced positive bands on larvae samples from *T. pseudospiralis* and *T. nativa* (Fig. 2). The next rounds of PCR analyses were performed to find out whether it was possible to design primers specific for *T. pseudospiralis* and *T. nativa.* We used the same reverse primer (primer set No. 2) which worked well for the first PCR round. The PCR products representing partial sequences of *T. pseudospiralis* and *T. nativa β‐CA* gene were sequenced, and the forward primers were designed based on the obtained sequences. These primers were included in the sets Nos. 5–7 for *T. pseudospiralis* and 8–11 for *T. nativa*, and the PCR results are shown in Fig. 3. Most primers produced multiple bands. When the most promising primers (No. 6 for *T. pseudospiralis* and No. 10 for *T. nativa*) were tested against all three species of *Trichinella*, they completely cross‐amplified because of the high sequence similarity (data not shown). Due to the interspecies cross‐amplification we further designed four new reverse primers which were used together with the previous forward primers resulting in primer set Nos. 12–16.

**Figure 4:**
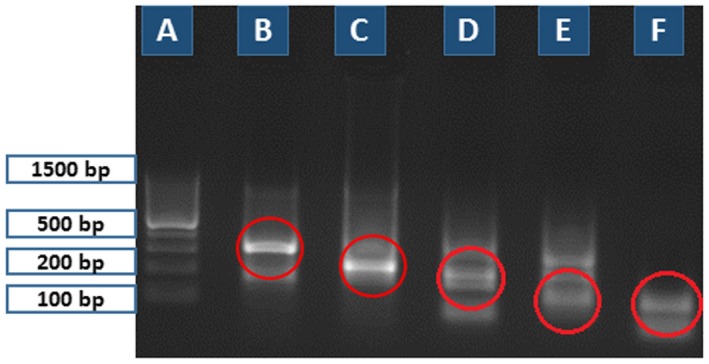
Third round of PCR tested on *T* *. nativa* and *T* *. pseudospiralis* samples (10 larvae/each lane). Primer set No. 12 for *T* *. pseudospiralis* and primer set Nos. 13–16 for *T* *. nativa*. (A) 100 bp ladder; (B) primer set No. 13 (319 bp), (C) primer set No. 14 (171 bp), (D) primer set No. 15 (168 bp), (E) primer set No. 16 (129 bp) for *T* *. nativa*, and (F) primer set No. 12 (116 bp) for *T* *. pseudospiralis*. The relevant PCR products are shown in red circles.

**Figure 5:**
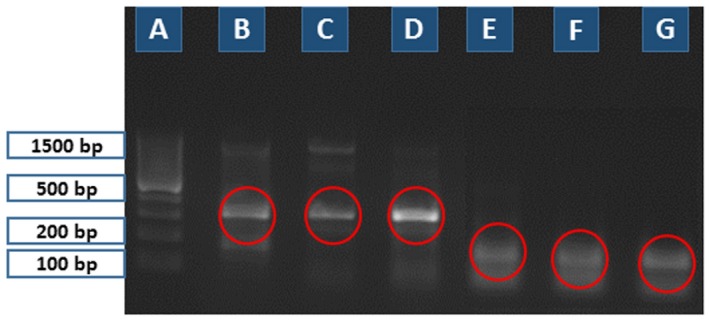
Evaluation of possible cross‐amplification between the primers designed for *T* *. nativa* and *T* *. pseudospiralis* β‐CA genes and templates from *T. spiralis*, *T. nativa*, and *T* *. pseudospiralis* (10 larvae/each lane). (A) 100 bp ladder; tested primer set No. 13 (product size 319 bp) on *T* *. nativa* (B), *T* *. pseudospiralis* (C), and *T* *. spiralis* (D); and primer set No. 12 (product size 116 bp) on *T* *. nativa* (E), *T* *. pseudospiralis* (F), and *T* *. spiralis* (G).

**Figure 6:**
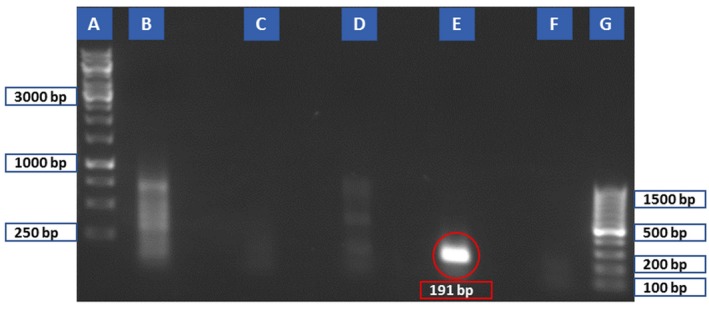
Evaluation of primer set No. 2 on different lysed parasites. (A) 1 kb ladder, (B) *T* *. gondii*, (C) *T* *. cati*, (D) *P* *. equorum*, (E) *T* *. spiralis* as the positive control, (F) negative control, and (G) 100 bp ladder. Red colour circle shows 191 bp PCR product of primer set No. 2 on *T* *. spiralis*.

The results revealed that the primer set No. 13 from *T. nativa* and primer set No. 12 from *T. pseudospiralis* produced the strongest DNA bands on the agarose gel electrophoresis (Fig. 4). For evaluation of cross‐amplification these primers were tested on all three *Trichinella* spp. Figure 5 shows that there was again a complete cross‐amplification between different *Trichinella* spp. by these primers. This finding led us to conclude that finding of species‐specific primers for *Trichinella β‐CA* genes is probably impossible due to the high sequence similarity. Based on our results the primer set No. 2 was considered the most potential tool for diagnostic purposes, even though it was not able to discriminate between different *Trichinella* spp. Therefore, its specificity was further tested on prepared samples from *Toxoplasma gondii*, *T* *oxocara cati*, and *P* *arascaris equorum*. No false‐positive reactions were detectable (Fig. 6).

## Discussion

Many protozoan, arthropod, and nematode species (invertebrate metazoans) contain *β‐CA* gene and protein sequences in their genomes and proteomes respectively. BLAST homology analysis of predefined β‐CA protein sequences from *D. melanogaster* (Uniprot ID: Q9VHJ5) and *C. elegans* (Uniprot ID: Q22460) resulted in identification of more than 75 β‐CA protein sequences from protozoans and invertebrate metazoans (Zolfaghari Emameh *et al*., 2014). One of them was β‐CA protein sequence from *T. spiralis* (UniProt ID: E5SH53). Our previous results also revealed that β‐CA proteins show high similarity among various *Drosophila* species and other species of dipteran flies (Zolfaghari Emameh *et al*., 2014). This led us to hypothesize that the high similarity is extensible to other species of one defined genus, such as *Trichinella* spp. including the clinically most relevant species: *T. spiralis*, *T. pseudospiralis*, and *T. nativa*. In principle, the high sequence homology at gene level could provide a straightforward avenue to develop a genus‐specific PCR assay for diagnostics of *Trichinella* infection (trichinellosis). On the other hand, the high sequence similarity could be a difficult challenge for development of a species‐specific assay based on the *β‐CA* gene.

A number of PCR‐based diagnostic methods have been developed for detection of infectious diseases, and some of them have been applied for parasitic diseases, such as trichinellosis (Li *et al*., 2010). In our study, we designed and tested 16 different pairs of primers for detection of β‐CA genomic sequences of *Trichinella* spp. At the beginning of assay development, it is necessary to test the efficiency of designed primers. Our method using the primer set No. 2 seemed to detect minute amounts of DNA (a single larva/reaction). Thereafter, the set No. 2 was considered the most promising primer pair for the development of genus‐specific tool for *Trichinella* diagnostics. The results also revealed that interspecies cross‐amplification is a recurrent challenge in this assay system when the DNA sequences are highly similar. None of the tested primers were able to discriminate between different *Trichinella* spp. As already pointed out, the described method was no able to discriminate between various species of *Trichinella*. This same problem hampers most detection methods reported to date. All species and genotypes of the genus *Trichinella* are morphologically indistinguishable at all developmental stages. Consequently, only biochemical or molecular methods can be used to identify the genotype of the parasite (Gajadhar *et al*., 2009).

Our results showed that the developed assay is robust enough for screening of *Trichinella* infection in routine meat samples. The positive signals were genus‐specific since no false‐positive bands were detectable when samples from other parasites, a protozoan *T. gondii* and two nematodes *T. cati* and *P. equorum*, were used as templates. Importantly, no *β‐CA* genes are present in mammalian genomes (Syrjanen *et al*., 2010; Zolfaghari Emameh *et al*., 2014). Therefore, host‐derived false‐positive reactions would be unlikely. Other advantages of the present method include both ease of use and rapidity of detection. The meat samples are lysed overnight, and thereafter, the sample is ready for PCR amplification. No separate DNA isolation procedure is needed for the preparation of the template. The PCR reaction and gel electrophoresis take about 3 h after which the positive bands are clearly visible for documentation.

As an overall conclusion, even though there are many diagnostic methods available for trichinellosis, most of them are either expensive, time‐consuming or require special expertise. Therefore, new simple tools are urgently needed for screening of biological samples in livestock slaughterhouses and quality controlling laboratories of meat processing companies. Our developed method is based on detection of β‐CA genomic sequences from *Trichinella* spp. The greatest advantage of this method is that mammalian hosts do not contain any *β‐CA* gene sequences in their genomes. The results indicated that the PCR method is *Trichinella* genus‐specific and robust enough for routine screening purposes.

## Experimental procedures

### Identification of β‐CA protein sequence from *T* *. spiralis*

β‐CA protein sequence identification number (ID: E5SH53) of *Trichinella spiralis* in UniProt database (http://www.uniprot.org/) was retrieved by BLAST homology analysis using the previously defined β‐CA protein sequences from *D. melanogaster* (Uniprot ID: Q9VHJ5) and *C. elegans* (Uniprot ID: Q22460) (Pearson, 2014; Zolfaghari Emameh *et al*., 2014). The corresponding genomic sequence ID of *T. spiralis* (EFV55868) was obtained from Ensembl Metazoa (http://metazoa.ensembl.org/index.html). Moreover, MSA by Clustal Omega algorithm (Sievers *et al*., 2011) within EMBL‐EBI database (http://www.ebi.ac.uk/Tools/msa/clustalo/) was applied to approve the blast homology result. MSA revealed highly conserved sequences within β‐CA protein sequences from *T. spiralis*, *D. melanogaster*, and *C. elegans*.

### Preparation of parasites and infected meat samples

Parasite samples and *Trichinella* spp.‐infected mouse muscle specimens were prepared at the Department of Veterinary Biosciences, University of Helsinki, Finland. The Animal Experiment Board in Finland had approved the study protocol. The tested *Trichinella* spp. involved *T. spiralis*, *T. pseudospiralis* and *T. nativa*. The blinded samples, including both infected and uninfected meat, were packed and shipped to Tissue Biology group, School of Medicine, University of Tampere, Finland, for PCR analysis. After arriving in the laboratory, the samples were transferred to −20°C freezer. To test the specificity of different primers, three more parasite samples were analysed including *T. gondii* (in culture medium), *T. cati* and *P. equorum* (in 70% ethanol).

### Lysis of the parasites and meat samples

The meat and larvae samples were handled under the laminar hood. *Toxoplasma gondii* parasites were separated from the culture medium by centrifugation (Heraeus Biofuge Fresco, Thermo Scientific, Waltham, MA) at 13 000 RPM for 10 min (Leroux *et al*., 2015). Then the following lysis protocol was performed on all the samples: 300 μl of lysis buffer (DirectPCR Tail Lysis reagent, Viagen Biotech, LA) was added to 5 mg of samples. Then 6 μl of proteinase K (Thermo Scientific) was added to the mixture. The lysis tubes were incubated in rotating incubator (HYBAID, Thermo Scientific) at 55°C overnight.

### Primer design

Three sets of primers for genomic sequence of β‐CA (exonic and intronic) from *T. spiralis* (Ensembl Metazoa ID: EFV55868) were designed automatically by NCBI Primer‐BLAST tool (http://www.ncbi.nlm.nih.gov/tools/primer‐blast/) (Ye *et al*., 2012), and one additional primer set was designed manually (Table 1). The expected sizes of the PCR products were calculated by Oligo Calc (http://www.basic.northwestern.edu/biotools/oligocalc.html) (Kibbe, 2007). To obtain genomic sequences for β‐CA of *T. pseudospiralis* and *T. nativa* we first performed PCR amplification using the designed *T. spiralis* primers, and *T. pseudospiralis* and *T. nativa* larvae were used as templates. The corresponding bands for *T. pseudospiralis* and *T. nativa* on the agarose gel were cut and sequenced as described in the sequencing section. The obtained β‐CA genomic sequences from *T. pseudospiralis* and *T. nativa* were aligned by Clustal Omega algorithm (http://www.ebi.ac.uk/Tools/msa/clustalo/) (Sievers *et al*., 2011) within EMBL‐EBI database (http://www.ebi.ac.uk/Tools/msa/clustalo/) against the genomic sequence of β‐CA from *T. spiralis*, and the most non‐identical regions were selected for the second round of primer design. We designed 3 and 4 different forward primers manually for *T. pseudospiralis* (primer set Nos. 5–7) and *T. nativa* (primer set Nos. 8–11), respectively, and used with the same reverse primer No. 2 which was described for the first round. In the third round of primer design, one new reverse primer was designed for *T. pseudospiralis* (primer set No. 12) and used with its own forward primer No. 7. Also, four new reverse primers were designed for *T. nativa* and tested with its own forward primers Nos. 8 and 10 (primer set Nos. 13–16). Three other species, *T. gondii*, *T. cati*, and *P. equorum* were tested for any false‐positive cross‐amplifications with other parasites.

**Table 1:** Designed primers for *β‐* *CA* genomic sequences from *T* *. spiralis*, *T* *. pseudospiralis* and *T* *. nativa.*

No.	Designed primers	Species name	Product length (bp)
**1**	**Forward:** 5′‐AGAGACTGCCCGTTCACAAG‐3′ **Reverse:** 3′‐CTGGGAGAGTTTGTCAGCGT‐5′	*T. spiralis*	882
**2**	**Forward:** 5′‐TTTGAGCGCACTAGCATCCA‐3′ **Reverse:** 3′‐TCCATTCTGCATCACGCTGT‐5′	*T. spiralis*	191
**3**	**Forward:** 5′‐AGACTGCCCGTTCACAAGTT‐3′ **Reverse:** 3′‐TGGATGCTAGTGCGCTCAAA‐5′	*T. spiralis*	530
**4**	**Forward:** 5′‐ATTAATAAGTGAAAAGCACA‐3′ **Reverse:** 3′‐GTTTTAGAACTGGACACTGT‐5′	*T. spiralis*	83
**5**	**Forward:** 5′‐CTCACCCATCACCCCGGCTT‐3′ **Reverse:** 3′‐TCCATTCTGCATCACGCTGT‐5′	*T. pseudospiralis*	251
**6**	**Forward:** 5′‐GGCCAGGCCGTTCATCTGGT‐3′ **Reverse:** 3′‐TCCATTCTGCATCACGCTGT‐5′	*T. pseudospiralis*	158
**7**	**Forward:** 5′‐ATCGCGCTCTCGCGATTGGG‐3′ **Reverse:** 3′‐TCCATTCTGCATCACGCTGT‐5′	*T. pseudospiralis*	103
**8**	**Forward:** 5′‐TCCCAGACCAGCGGNAGCAC‐3′ **Reverse:** 3′‐TCCATTCTGCATCACGCTGT‐5′	*T. nativa*	372
**9**	**Forward:** 5′‐GACCCAGCGCGCTTTCGTTG‐3′ **Reverse:** 3′‐TCCATTCTGCATCACGCTGT‐5′	*T. nativa*	317
**10**	**Forward:** 5′‐CGGATACCACGGGCCGATGT‐3′ **Reverse:** 3′‐TCCATTCTGCATCACGCTGT‐5′	*T. nativa*	226
**11**	**Forward:** 5′‐AGTCGCCCAGCTTGATCGCG‐3′ **Reverse:** 3′‐TCCATTCTGCATCACGCTGT‐5′	*T. nativa*	116
**12**	**Forward:** 5′‐ATCGCGCTCTCGCGATTGGG‐3′ **Reverse:** 3′‐CAACCGATACCGAACGGACC‐5′	*T. pseudospiralis*	116
**13**	**Forward:** 5′‐TCCCAGACCAGCGGNAGCAC‐3′ **Reverse:** 3′‐CGGACCGAACTCTGGTACAG‐5′	*T. nativa*	319
**14**	**Forward:** 5′‐CGGATACCACGGGCCGATGT‐3′ **Reverse:** 3′‐CGGACCGAACTCTGGTACAG‐5′	*T. nativa*	171
**15**	**Forward:** 5′‐TCCCAGACCAGCGGNAGCAC‐3′ **Reverse:** 3′‐ACATCGGCCCGTGGTATCCG‐5′	*T. nativa*	168
**16**	**Forward:** 5′‐TCCCAGACCAGCGGNAGCAC‐3′ **Reverse:** 3′‐GTGAGTCCAGCAGCAACCCG‐5′	*T. nativa*	129

### PCR

PCR was performed based on the following reaction mixture: 12.5 μl of 2X KAPA ReadyMix (KAPA 2G Robust HotStart ReadyMix PCR Kit, Kapa Biosystems, Wilmington, MA), 1.25 μl of forward primer (Oligomer, Helsinki, Finland), 1.25 μl of reverse primer (Oligomer), 9 μl of dH_2_O and 1 μl of lysed samples. Lysed *T. spiralis* larvae served as a positive control and the negative control was the PCR master mix without any lysed sample. The PCR assay was run on the thermocycler (PTC100, MJ Research Inc, Waltham, MA) according to the following details: 95°C (3 min), [95°C (10 s), 53°C (10 s), 72°C (10 s)] × 36 cycles, 72°C (5 min).

### Sequencing of β‐CA genomic sequences from *T* *. pseudospiralis* and *T* *. nativa*

We cut the PCR products of *T. pseudospiralis* and *T. nativa* templates from an agarose gel. Illustra‐GFX PCR DNA and Gel Band Purification Kit (GE Healthcare, Buckinghamshire, UK) was applied for extraction and purification of the corresponding DNA bands. At the final step, the overnight‐dried samples were suspended in HiDi (Life Technologies Europe, Applied Biosystems, Finland), which was followed by vortexing and spinning. The samples were heated at 95°C for 2 min and transferred to ice for 2 min. The samples were sent to Core Facilities and Research Services, BioMediTech, Tampere, Finland for DNA sequencing. The sequencing was carried out with HITACHI 3130x/Genetic Analyzer (Life Technologies Europe, Applied Biosystems).

## Authors' contributions

RZE carried out blast homology analysis and MSA on *T. spiralis*, *T. pseudospiralis*, *T. nativa*, *C. elegans*, and *D. melanogaster* β‐CA sequences. RZE and MK participated in the primers design and setting up the PCR method. AN and AS prepared the parasite and infected meat samples. All authors participated in the design of the study. RZE and MK drafted the first version of the paper. All authors read and approved the final manuscript.

## Conflict of Interest

None declared.
